# Measuring Environmental Health Literacy – A Systematic Review of Represented Dimensions and Psychometric Characteristics of Evaluated Instruments in an Emerging Field

**DOI:** 10.1177/11786302261462601

**Published:** 2026-08-04

**Authors:** Miriam Finkhäuser, Renée Madelaine Kuhrt, Marlene Muehlmann, Tobias Ihle, Silke Schmidt, Holger Muehlan

**Affiliations:** 1 Department Health and Prevention, Institute of Psychology, University of Greifswald, Greifswald, Germany; 2 German Center for Child and Adolescent Health (DZKJ), partner site Greifswald/Rostock, Greifswald, Germany; 3 Department of Medicine, Division Medical Psychology & Medical Sociology, Health & Medical University Erfurt, Erfurt, Germany

**Keywords:** environmental health literacy (EHL), health literacy, public health tools, environmental exposure, health risk communication

## Abstract

Environmental health literacy (EHL) encompasses the skills and competencies to search for, understand, assess, and apply information and knowledge about the environment and its impact on human health. The development and availability of concept-based, psychometrically sound methods to measure EHL are crucial for advancing research and assessing the impact of public health programs in prevention. Guided by the PRISMA-COSMIN guidelines, a systematic search of three databases was conducted (MEDLINE, LIVIVO, PsycINFO). Information on the characteristics, operationalization of EHL, and psychometric criteria was extracted. According to COSMIN, the psychometric properties were evaluated. Of 1,872 records, nine studies were included. Eight different EHL measures were identified. The instruments target either general EHL (n = 4) or specific environmental issues (n = 4), such as air pollution, drinking water contaminants, and phthalate exposure. Some instruments reported sufficient structural validity (n = 4) and internal consistency (n = 2), while content validity remained indeterminate (n = 8). Information on responsiveness, measurement error, and intercultural validity could not be obtained. Based on the quality assessment, no general recommendation for use can be derived. Future studies should focus on validating existing instruments, while the development of new tools should more comprehensively address relevant environmental factors. We also recommend that previously underexplored dimensions of EHL, such as self-efficacy and collective agency, should be given greater consideration.

## 1. Introduction

Growing pressure on the global environment, along with environmental challenges, poses significant threats to human health.^1,2^ The World Health Organization (WHO) estimates that approximately 24% of global deaths are attributable to modifiable environmental factors.^3^ Major risk factors include air pollution, unsafe water and hygiene conditions, and chemical pollution – all linked to serious health conditions.^4^ In this context, the ability to make informed health decisions relies on individuals’ capacity to access, understand, evaluate, and apply information about environmental risks—a competency known as Environmental Health Literacy (EHL).^5-7^

EHL integrates elements from health literacy (HL), environmental literacy (EL), environmental justice, and environmental and health sciences.^5,8^ HL refers to the ability to access, understand, and use health information to make informed decisions.^9,10^ This includes interpreting medical instructions, following doctors’ advice, and navigating healthcare systems.^11^ EL, first introduced by Charles Roth in 1968, involves recognizing, understanding, and addressing environmental issues.^12,13^ United Nations Educational, Scientific, and Cultural Organization (UNESCO) later advanced environmental literacy through global environmental education initiatives.^14^ EHL also has its origins in the environmental justice movement, highlighting how marginalized communities face higher exposure to pollution and related health risks.^8,15^ Expanding on HL and EL, EHL emphasizes the environmental factors affecting health.^6^ Its goal is to raise awareness of environmental risks and develop strategies to reduce health hazards.^5^

A model by Finn and O'Fallon^5^ and another by Gray^6^ emphasize that EHL goes beyond mere knowledge and requires its active application in everyday life. Finn and O'Fallon^5^ describe EHL as a dynamic, multi-stage process with varying levels of competence. Drawing from Bloom’s taxonomy,^16^ they introduce EHL as a hierarchy of skills, from basic hazard recognition to the ability to develop independent solutions. This model underscores EHL’s non-linear, context-dependent nature and serves as a foundation for education and research, particularly in vulnerable communities.^5^

Gray^6^ presents a three-dimensional model of EHL, where each dimension builds on the previous one. The first dimension involves awareness and knowledge of how environmental pollution affects health. The second focuses on skills and self-efficacy for making health-protective decisions— referring to an individual’s confidence in their ability to take effective action to reduce harmful environmental impact. The third emphasizes community change, highlighting the need to apply knowledge and skills within a community to reduce exposure effectively.

Despite several models and various definitions, no consensus exists on the essential skills for EHL.^8^ Its operationalization is diverse, complicating the development of standardized measurement tools.^7^ The complexity increases due to the wide range of environmental factors affecting health, making comprehensive assessment challenging.^6,7^ Gray^6^ highlights the use of qualitative methods, such as focus groups and interviews, to explore public understanding of environmental health risks, and the use of pre-/post-designs to evaluate changes in knowledge and behavior after interventions. Pfleger et al.^7^ note a high degree of variability in EHL measurement approaches, with most studies relying on questionnaires. However, comparisons are difficult due to methodological inconsistencies regarding the operationalization of EHL.

To our knowledge, no review has systematically evaluated the psychometric quality of instruments measuring EHL. Previous reviews^6,7^ have summarized existing approaches but did not assess their measurement properties. This systematic review, therefore, aims to identify available instruments for measuring EHL and to provide an overview of their psychometric quality to inform the selection of suitable measurement approaches.

The key questions addressed in this review are as follows:

• What instruments are available to assess EHL?
• How is EHL operationalized, and which dimensions are captured by these instruments?
• What is the psychometric quality of the existing instruments?

## 2. Materials and Methods

This systematic review was conducted following the PRISMA-COSMIN guideline, specifically developed for preparing and reporting systematic reviews on outcome measurement instruments in health research,^17^ which integrates the Preferred Reporting Items for Systematic Reviews and Meta-Analyses framework (PRISMA)^18^ and the Consensus-based Standards for the selection of health Measurement Instruments (COSMIN) Initiative.^19^ We provide detailed supplementary material for the search string (Additional File Table S 1), pre-defined inclusion and exclusion criteria (Additional File Table S 2), and the quality assessment for good measurement properties (Additional File Table S 5 and Table S 6).

### 2.1. Systematic Search

We searched LIVIVO, MEDLINE, and APA PsycInfo on 12 September 2024. The strategy combined two concepts using the Boolean operator AND: (1) “environmental health literacy” and related literacy terms (e.g., knowledge, education), and (2) measurement terms (e.g., instrument, tool, survey, scale, assessment, measurement, inventory, questionnaire, interview). German synonyms were searched in a parallel string.

### 2.2. Inclusion and Exclusion Criteria

Study selection followed predefined inclusion and exclusion criteria. We included primary studies in German or English that reported the development and/or validation of an EHL instrument and provided sufficient detail on psychometric properties. At a minimum, studies had to report content validity and internal structure (structural validity and internal consistency). We excluded studies that did not explicitly measure EHL (e.g., general health literacy, environmental awareness, or knowledge of environmental risks), studies that used an EHL instrument without evaluating its measurement properties, and non-empirical publications (books, manuals, reviews, meta-analyses, dissertations, commentaries). Studies in other languages were excluded unless a German or English translation was available.

### 2.3. Selection of Studies

One author imported records from MEDLINE, LIVIVO, and PsycInfo. After removing duplicates in Zotero, the same author screened titles and abstracts against the predefined criteria mentioned above. Backward and forward citation tracking of included studies identified one additional record. Two raters assessed full texts; disagreements were resolved by discussion until consensus. One author extracted (i) study characteristics (authors, year, country, design, sample size, sample characteristics), (ii) instrument characteristics (name, development language, application context/environmental topic, EHL operationalization, measurement dimensions, number of items), and (iii) results on psychometric properties. Quality assessment was conducted by two raters following the COSMIN criteria for good measurement properties,^19^ adapted for the EHL context. Disagreements were resolved by discussion until a consensus was reached. Two raters performed a risk-of-bias assessment using the Checklist for the Quality of Quantitative Studies.^20^ Findings and appraisals were synthesized narratively, highlighting similarities, differences, and critical evaluations across studies and instruments.

## 3. Results

**Figure 1.**
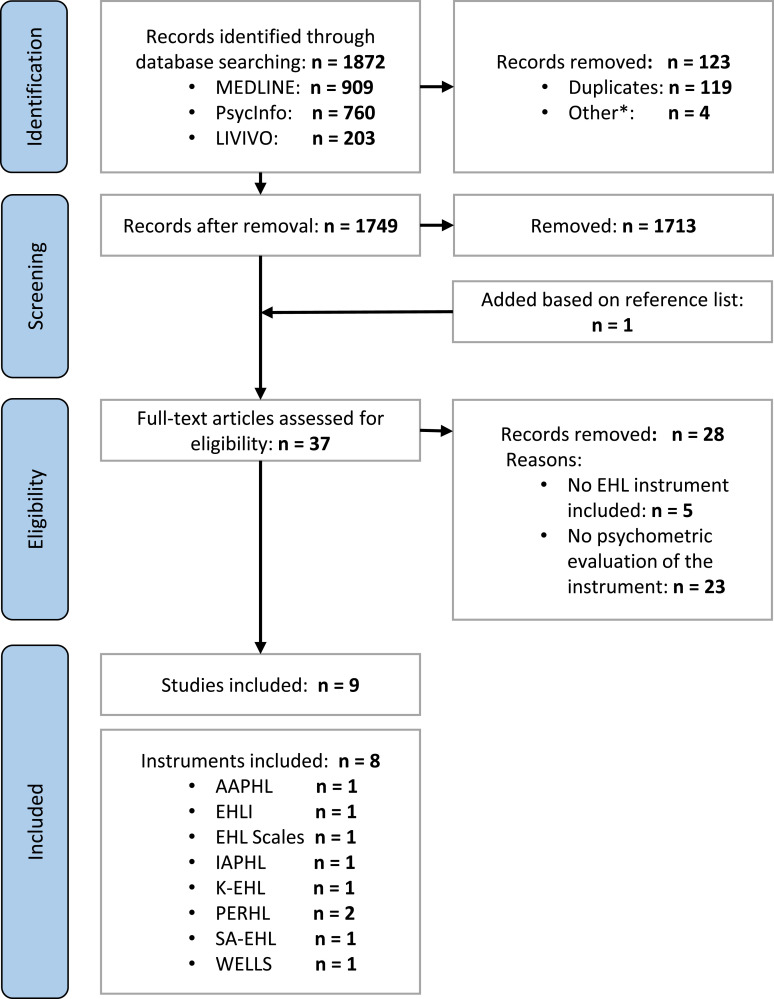
Flowchart of the study selection process Note. Flowchart is based on the PRISMA-COSMIN guidelines.^17^ AAPHL: Ambient Air Pollution Health Literacy Instrument, EHLI: Environmental Health Literacy Index, EHL Scales: Environmental Health Literacy Scales, IAPHL: Indoor Air Pollution Health Literacy Instrument, K-EHL: Korean version of the Environmental Health Literacy Scale, PERHL: Phthalate Environmental Reproductive Health Literacy Scale, SA-EHL: Short Assessment of Environmental Health Literacy, WELLS: Water Environmental Literacy Level Scale. Other*: These articles were withdrawn from publication.

The search identified 1,872 records (Figure 1). After removing four withdrawn MEDLINE records and 119 duplicates, 1,749 records remained for title and abstract screening. Of these, 36 were deemed eligible and retrieved for full-text review. Backward citation searching of reference lists yielded one additional article, which was excluded at full-text screening because it did not meet the inclusion criteria. In total, 37 full-text articles were assessed; nine studies were ultimately included, representing eight distinct EHL instruments.^21-30^ One instrument was examined in two studies^28,30^—one reporting its development and the other evaluating its construct validity. Five studies were excluded because no specific instrument for EHL was developed or validated, and n = 23 were excluded because they provided insufficient information on quality criteria or lacked psychometric evaluation (detailed information in Additional File Table S7). The final dataset resulted from nine studies.^21-30^ Risk of Bias ranged from 1 to 2 (means in Table 1; detailed information in Additional File Table S8), with the highest risk of bias for the WELLS Study^23^ and the lowest for the K-EHL Study.^24^

**Table 1.** Overview of the Included Studies

Study	Country	Study design	Sample size	Sample characteristics	Risk of bias mean
Chen et al. (2023)	Taiwan	Cross-sectional study, telephone survey	*N* = 1297, *n* = 1072 via landline (response rate: 26.3 %), *n* = 225 via mobile phone (response rate: 27.0 %)	Age: 20 – 70 years, *M* = 46.9, *SD* = 13.9; Gender: 55.6 %; Female Level of education: 61 %; College degree or higher	1.9
Fiore et al. (2024)	Italy	Cross-sectional survey, paper questionnaire	*N* = 4778 students	Age: *M* = 21 years, *SD* = 4.3; Gender: 65.1 %; Female Study programs: 53.2 % natural science- and health-related, 46.8 % humanities and social sciences	1.7
Irvin et al. (2019)	USA	Cross-sectional study, online survey Mechanical Turk platform	*N* = 869 (response rate: 95.0 %; out of 911 who started the survey)	Age: *M* = 37.8 years, *SD* = 11.6; Gender: 57.5 % Female, 1 % not specified; Level of education: 51.8 % College degree, 12.5 % High school diploma	1.0
Kwak & Kim (2022)	South Korea	Methodological validation study, online survey	*N* = 492	Age: 19 – 65 years, *M* = 38.97, *SD* = 10.78, Gender: 50.6 %, Female Level of education: 85.8 % University degree or higher	2.0
Lichtveld et al. (2019)	USA	Cross-sectional study with two samples for exploratory and confirmatory factor analysis	Exploratory sample: *n* = 174 students of public health, Test sample: n = 98 Community members	Exploratory sample: Age: *M* = 23.8 years Gender: 77 % Female Education: 61 % Bachelor’s degree or higher	1.7
				Test sample: Age: *M* = 43.8 years Gender: 82 % Female Education: 49 % Bachelor’s degree or higher	
Rohlman et al. (2022)	USA	Cross-sectional study, online survey via the Amazon Mechanical Turk platform	*N* = 869, Response rate = 95 % (out of 911 who started the survey)	Age: *M* = 38 years, *SD* = 11.6 Gender: 57.9 % Female, <1 % none Specification Level of education: 52 % College degree, 12.4 % High school diploma	1.9
Tomsho, Quinn, Adamkiewicz, et al. (2024)	USA	Cross-sectional study, online survey	*N* = 117 participants from the ERGO cohort *(Environmental Reproductive and Glucose Outcomes)* who had given birth in the last five years	Age: *M* = 33.1 years, *SD* = 4.26; Level of education: 86.3 % University degree or higher	1.9
Tomsho, Quinn, Preston, et al. (2024)	USA	Cross-sectional survey, biomarkers	*N* = 117 participants from the ERGO cohort, of which n = 42 for urine sample analysis	Urine sample spot check: Age: *M* = 34.0 years, *SD* = 4.40; Level of education: 88.1 % University degree or higher	1.7
Wu et al. (2022)	Taiwan	Cross-sectional study, mixed-methods design	*N* = 647 (quantitative main study)	Age: *M* = 44.5 years, *SD* = 18.31; Gender: 52.7 % Female; Education level: 71.8 % College degree or higher	1.8

### 3.1. Characteristics of Included Studies

All included studies were published from 2019 to 2024. Two were conducted in Taiwan, five in the United States, and one each in Italy and South Korea (Table 1). Of the nine included studies, eight developed EHL instruments, and one examined the association between an EHL measure and biomarkers of chemical exposure. Table 2 summarizes the eight survey instruments: four assess general EHL, and four focus on specific environmental issues, including air pollution, contaminants in drinking water (arsenic), and phthalate exposure (Phthalates are endocrine disruptors found in plastics and personal care products. They can cause adverse pregnancy outcomes such as gestational diabetes, hypertension, and childhood obesity^28^).

**Table 2.** Overview of the Included Survey Instruments

Instrument, Study	Language	Environmental topic and intended use	Operationalization of the EHL	Measurement dimensions, number of items	Response options
Ambient Air Pollution Health Literacy Instrument (AAPHL)^21^	Chinese	Individual competencies related to *ambient air pollution*	Based on the integrated model of *Health Literacy (HLS-EU-Q)*; includes four information processing competences (access, understanding, appraise, application) in three health contexts (healthcare, disease prevention, health promotion)	12 dimensions (4 competences × 3 domains), 2 items each	4-point Likert scale (“very difficult” to “very easy”)
		Aim: to assist stakeholders in identifying public deficiencies/capacities/needs to better design targeted interventions		Total: 24 items	
Environmental Health Literacy Index (EHLI)^22^	Italian	*General EHL* among university students	Based on the *Health Literacy* Model according to Nutbeam (2000): *functional* EHL (importance of behaviours to combat environmental pollution), *interactive* EHL (agreement with statements about the connection between environmental pollu-tion and health), *critical* EHL (perception of health risks)	3 dimensions: functional (6 items), critical (4 items), interactive (3 items)	6-point Likert scale (“not important” to “extremely important”) functional and critical EHL; 5-point Likert scale (“strongly agree” to “disagree”) for interactive EHL
				Total: 13 items	
Environmental Health Literacy (EHL Scales)^25^	English	Diverse community contexts: Air pollution, water quality, food contamination and general environmental health	Knowledge, attitudes and behaviour in relation to environmental media such as air, water and food, as well as general environmental health	4 scales with 3 dimensions each: knowledge, attitudes and behaviour	5-point Likert scales, (“strongly agree” to “strongly disagree”) for knowledge and attitude items; frequency of behaviour for behavioural items
				Total: 42 items	
Indoor Air Pollution Health Literacy Instrument (IAPHL)^29^	Chinese	For researchers and stakeholders to measure individuals' competencies regarding *indoor air pollution*	Based on the integrated model of *Health Literacy (HLS-EU-Q)*; includes four information processing competences (access, understanding, evaluation, application) in three health contexts (healthcare, disease prevention, health promotion)	12 dimensions (4 competences × 3 domains)	4-point Likert scale (“very difficult” to “very easy”)
				Total: 38 items	
Korean version of the Environmental Health Literacy Scale (K-EHL)^24^	Korean	*Air pollution, water quality, food contamination* and *general EHL* for Korean context	Based on the original scale from Lichtveld et al. (2019); knowledge, attitudes and behaviour in relation to environmental media such as air, water and food, as well as general environmental health	2 dimensions: EH knowledge and attitude (14 items), EH behaviour (24 items)	5-point Likert scale
				Total: 38 items	
Environmental Reproductive Health Literacy Scale (PERHL)^28,30^	English	*Phthalates*-specific EHL in people of reproductive age (e.g., pregnant individuals)	Multidimensional scale for recording awareness, perceived uncertainty, protective behaviour, regulatory interest and knowledge in the context of phthalates in people of reproductive age	6 dimensions, 26 items (final version)	5-point Likert scale (“strongly disagree” to “strongly agree”)
Water Environmental Literacy Level Scale (WELLS)^23^	English	Frequent environmental pollutants in drinking water from private wells	Numerical skills and decision making in the context of well water quality, based on the *Newest Vital Sign* (NVS) *Health Literacy* Test	One-dimensional, 6 items	Multiple-choice and open questions, one point per correct answer, scores from 0 to 6

*Note.* HLS-EU-Q: European Health Literacy Survey Questionnaire. EH: Environmental Health.

Lichtveld et al.^25^ developed four scales: one for general EHL and three for specific environmental media covering air pollution, water quality, and food contamination. Specific environmental media refer to areas containing pollutants affecting individuals or communities.^25^ Kwak and Kim^24^ later adapted and translated these scales into Korean.

### 3.2. Operationalization of EHL

Six of the eight identified EHL measurement approaches rely on self-assessment questionnaires, while two—the Short Assessment of Environmental Health Literacy (SA-EHL) and the Water Environmental Literacy Level Scale (WELLS)**—**use objective measurement methods. The SA-EHL, developed by Rohlman et al.,^26^ is based on the Short Assessment of Health Literacy (SAHL)^31^ and measures the ability to recognize and understand environmental health terms by distinguishing between correct and distractor terms. The WELLS, created by Irvin et al.^23^ and modeled after the Newest Vital Sign (NVS) test,^27,32^ assesses calculation skills and decision-making related to water quality, particularly arsenic pollution.

In addition to the SA-EHL and WELLS, three further instruments build on HL frameworks. The Ambient Air Pollution Health Literacy (AAPHL) instrument by Chen et al.^21^ and the Indoor Air Pollution Health Literacy (IAPHL) instrument by Wu et al.^29^ are based on the European Health Literacy Survey Questionnaire (HLS-EU-Q) and evaluate four core skills: access to information, understanding, evaluation, and application. These skills are assessed in three health contexts: healthcare, disease prevention, and health promotion. The Environmental Health Literacy Index (EHLI) by Fiore et al.^22^ follows Nutbeam’s^33^ health literacy model, measuring three dimensions of EHL: functional EHL, defined as the subjective assessment of the importance of behaviors to reduce environmental pollution; interactive EHL, which evaluates agreement with statements about pollution-health links; and critical EHL, which assesses risk perception in the context of environmental pollution.

The remaining three instruments focus on measuring EHL through knowledge, attitudes, and behaviors. The Environmental Health Literacy Scales (EHL Scales) by Lichtveld et al.^25^ assess these dimensions across air, water, food, and general environmental health. The Korean Environmental Health Literacy Scale (K-EHL**)** by Kwak and Kim^24^ follows the same framework but combines knowledge and attitudes based on exploratory factor analysis. The Phthalate Environmental Reproductive Health Literacy (PERHL) scale, developed by Tomsho et al.,^28^ assesses knowledge, risk perception, protective behaviors, and perceived uncertainty about exposure to phthalates and their effects on reproductive health. Additionally, it includes a dimension measuring attitudes toward the regulation of phthalates. Across all instruments, higher total scores indicate greater EHL.

### 3.3. Description and Rating of Psychometric Quality Criteria of the Instruments

#### 3.3.1. Content Validity

Based on the relevance, comprehensiveness, and comprehensibility ratings, the overall content validity for all eight instruments was rated as indeterminate (Figure 3).

**Figure 2.**
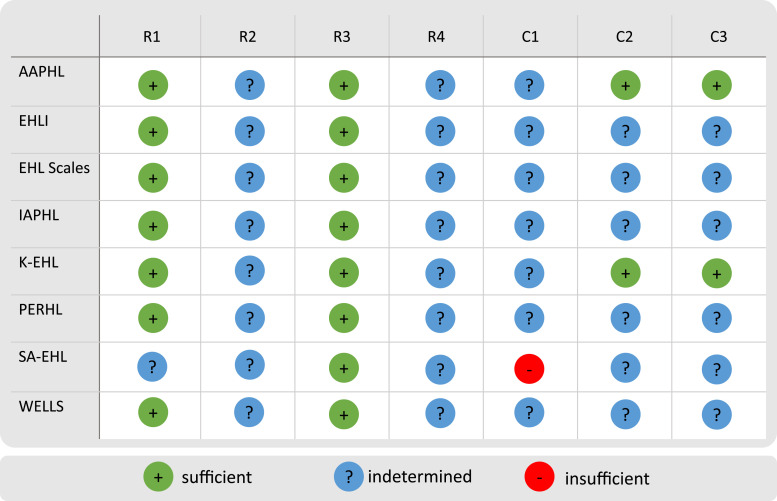
Results of the content validity assessment Note. R1-R4 – relevance; C1 – comprehensiveness; C2 and C3 – comprehensibility. An overview of the assessment criteria and the requirements can be found in the supplements (Additional File Table S 5 and Table S 6)

The relevance and comprehensiveness assessment examines whether the instruments capture all relevant aspects of the measured construct (Figure 2). Across all instruments, experts were involved in the development or selection of items, indicating that the items can be considered relevant for the construct being measured. For instance, three instruments (AAPHL, K-EHL, and IAPHL) used the Content Validity Index (CVI), a quantitative method in which professionals evaluate how well items reflect the intended construct. CVI values range from 0 to 1, with higher values indicating better alignment. However, none of the included instruments involved a representative sample of the target population in the concept elicitation, and essential information for assessing all criteria within the “relevance” category is missing. As a result, the overall rating for relevance across all eight instruments is classified as indeterminate.

To ensure comprehensiveness, conceptual saturation must be reached—meaning qualitative data collection continues until no new relevant insights emerge.^19^ This process often involves interviews or focus groups with participants from the target population. However, none of the eight included studies provides evidence that saturation was achieved during instrument development. Instead, the instruments were primarily derived from expert opinions, existing instruments, and literature rather than direct input from the target population**.** While the AAPHL^21^ and IAPHL^29^ instruments underwent three phases of cognitive interviews to gather participant feedback, there is no indication that development was concluded on the basis of an absence of new findings. Consequently, it remains indeterminate whether all essential dimensions of EHL were adequately represented in these instruments. The SA-EHL received an insufficient rating for comprehensiveness, as evidence suggests its 17 items do not fully capture EHL and may instead primarily assess word recognition.^26^

The comprehensibility assessment examines whether the target population understands the instructions (C2), items, and response options (C3) of the instruments as intended. Two included studies conducted pilot surveys and reported modifications based on participant feedback. For example, Chen et al.^21^ revised the AAPHL instrument to improve clarity by providing detailed explanations of legal references (e.g., the Air Pollution Control Act) and by eliminating double-negative sentences. Furthermore, Kwak and Kim^24^ adjusted the K-EHL instrument’s structure based on participant feedback, organizing items by knowledge, attitude, and behavior rather than environmental media, which likely improved clarity for respondents. However, the remaining studies did not provide sufficient information regarding efforts to enhance comprehensibility. For example, while Wu et al.^29^ describe a three-phase cognitive interview process to assess comprehension of the IAPHL instrument, they fail to clarify whether and how specific comprehension difficulties were addressed. Consequently, despite some evidence of efforts to ensure comprehensibility, reporting on this aspect remains incomplete and inconsistent.

#### 3.3.2 Structural Validity

**Figure 3.**
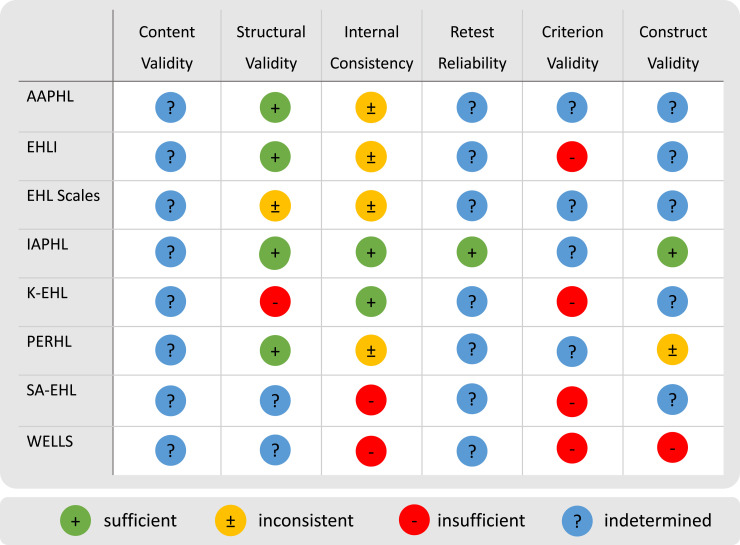
Results of the overall evaluation of the quality criteria Note. An overview of the evaluation criteria and the requirements for a “sufficient” (+) rating can be found in the supplements. The quality criteria intercultural validity, measurement error and responsiveness are not shown in this figure, as none of the included studies reported relevant information, thus precluding any corresponding quality assessment.

Four out of eight instruments – AAPHL,^21^ EHLI,^22^ IAPHL,^29^ and PERHL^28^ - demonstrated sufficient structural validity (see Figure 3 and File Table S3 for more details). The K-EHL scale^24^ received an insufficient rating, as it failed to meet both EFA and CFA criteria. For the SA-EHL^26^ and the WELLS^23^ information on factor loadings and explained variance was lacking, resulting in an indeterminate rating for both instruments.

The EHL scales by Lichtveld et al.^25^ received inconsistent ratings due to mixed results (Figure 3). Two of the four subscales, the food and water subscales, showed sufficient results in the exploratory factor analysis (EFA), but did not meet the criteria in the confirmatory factor analysis (CFA) (RMSEA > .06). In contrast, the air subscale and the general environmental health subscale had adequate RMSEA values (<.06) but performed insufficiently in the EFA (total variance explained < 50%). Due to these conflicting results, the overall structural validity of the EHL scales by Lichtveld et al.^25^ was rated as inconsistent. Further information on the structural validity of all instruments can be found in the supplements (Additional File Table S3).

#### 3.3.3. Internal Consistency

Regarding reliability, internal consistency was measured using Cronbach’s alpha in most studies, except for the PEHRL^28^ measure, for which the omega coefficient was used, a more accurate measure when item correlations with the construct vary. Internal consistency was rated as sufficient for the IAPHL instrument^29^ and the K-EHL scale,^24^ as both instruments met the Cronbach’s alpha threshold of > .70, including for the K-EHL subscales. In contrast, the SA-EHL (Cronbach’s α = .56) and WELLS (Cronbach’s α = .51) did not meet this criterion and were therefore rated as insufficient. The AAPHL instrument^21^ received an inconsistent rating. While the overall scale demonstrated high internal consistency (Cronbach’s α = .93), eight of the twelve individual dimensions had composite reliability values below .70. A similar issue was found in the EHLI,^22^ which showed sufficient internal consistency overall (Cronbach’s α = .81). However, its interactive EHL dimension did not meet the criterion (Cronbach’s α = .47). The EHL scales^25^ also only partially met the internal consistency criterion. While the air scale and the general EH scale met the threshold (Cronbach’s α = .70), the food and water scales fell short, with values of .67 and .63, respectively. The PERHL scale,^28^ was evaluated using the omega coefficient and received an inconsistent rating for internal consistency. Of its six dimensions, “Regulatory Interest” did not meet the criterion (ω = .63).

#### 3.3.4. Retest Reliability

Regarding retest reliability, data was available for only one instrument. The IAPHL instrument^29^ demonstrated sufficiently high test-retest reliability over 4 weeks, with an intraclass correlation coefficient (ICC) of .94.

#### 3.3.5. Criterion Validity

Criterion validity was examined in four studies by comparing EHL instruments to established measures of similar constructs. Fiore et al.^22^ assessed correlations among the EHLI, functional health literacy, and frequency of pro-environmental behavior. Kwak and Kim^24^ compared the K-EHL with the validated K-EHEP, a Korean adaptation of the Environmental Health Engagement Profile (EHEP).^34^ Rohlman et al.^26^ and Irvin et al.^23^ tested their SA-EHL and WELLS instruments against their source measures, the SAHL and NVS, respectively, which assess general health literacy.

However, all four studies were rated as insufficient, because none of the study results exceeded the threshold of .70 for correlations with another measurement instrument or for the area under the curve (AUC). In the study by Fiore et al.^22^ the correlation between the EHLI and functional health literacy was weak (Spearman’s ρ = .183), as was the correlation with pro-ecological behaviors (ρ = .248). Additionally, a receiver operating characteristic (ROC) curve was calculated to predict pro-ecological behavior, yielding an AUC of .643 (p < .001), suggesting limited discriminatory ability. In the study by Kwak and Kim,^24^ the correlation between K-EHL and K-EHEP was .67, which, although statistically significant, did not meet the >.70 criterion, resulting in an insufficient rating. Similarly, Rohlman et al.^26^ reported a low correlation between the SA-EHL and the SAHL (r = .11, p < .001), which also received an insufficient rating. In the study by Irvin et al.^23^ the correlation between the WELLS and the NVS was statistically significant but still below the required threshold (ρ = .47, p < .001), resulting in an insufficient rating.

#### 3.3.6. Construct Validity

Four of the nine included studies report construct validity results, with mixed findings. Two of these studies relate to the PERHL scale. Tomsho et al.^28^ investigated the relationship between scores on their developed instrument and self-reported avoidance of phthalate-containing products. Significant correlations were found for three of the six dimensions of the PERHL scale. Participants with higher scores in the dimensions “protective behavior/risk control” [odds ratio (OR) = 21.21 (95% confidence interval (CI): 6.16, 98.34)], “awareness of exposure pathways” [OR = 5.45 (95% CI: 1.57, 23.78)] and “general phthalate knowledge” [OR = 4.79 (95% CI: 1.26, 19.51)] were more likely to report avoiding phthalates in personal care products. In contrast, a higher PERHL total score was associated with a slightly lower likelihood of reporting phthalate avoidance [OR = 0.63 (95% CI: 0.41, 0.89)]. In a second study by Tomsho et al.,^30^ in addition to PERHL scale values, biomarkers of phthalate exposure were measured in urine samples. A significant correlation was found between higher PERHL scores and lower phthalate concentrations in urine samples. The authors concluded that a higher EHL could act as a potential protective factor against harmful environmental influences. Given partial confirmation of the hypothesis, construct validity was rated as inconsistent overall.

In the study by Irvin et al,^23^ the authors hypothesized that experience with either a well or septic system would positively influence EHL. Contrary to expectations, respondents with experience scored lower on both the WELLS and NVS. As a result, construct validity was rated as insufficient. In the study by Wu et al.,^29^ the discriminant validity of the IAPHL instrument was investigated. This represents a sub-form of construct validity and tests whether concepts or measurements that are theoretically unrelated actually show no correlation. The authors hypothesized that the IAPHL instrument scores would correlate only weakly with the number of hours spent watching TV per week, because news media in Taiwan rarely report on the health effects of indoor air pollution.^29^ The Spearman correlation coefficient supported this assumption, showing a weak association of ρ = -.14 (p < .001) between hours spent watching TV weekly and the IAPHL total score. This was rated as in line with the hypotheses and therefore classified as sufficient.

## 4. Discussion

This study aimed to review available measurement instruments for assessing EHL and evaluate their quality and suitability across different contexts. A systematic literature review identified nine studies covering eight instruments. These were assessed using the COSMIN criteria for good measurement properties,^19^ with structural validity and internal consistency receiving the most thorough analysis. The findings highlight both strengths and gaps in EHL measurement.

### 4.1. Identified Studies and Survey Approaches

The literature review revealed that all included studies were published from 2019 to 2024. This suggests that research on EHL is still emerging and that interest in developing measurement instruments is growing. Notably, half of the identified instruments were developed in the United States, three originated in Asia, and one originated in Europe. This uneven distribution aligns with findings by Pfleger et al.,^7^ who also identified a research gap in Europe, while studies from the U.S. dominated. One possible explanation is that key conceptual frameworks for EHL, such as those proposed by Finn and O'Fallon^5^ and Gray,^6^ originated in the U.S., laying the foundation for research and instrument development primarily in that country.

The identified instruments vary widely in scope. Half of the instruments target specific environmental issues, while two measure general EHL and two others integrate both general EHL and specific environmental factors such as air, water, and food. Certain pollutants, including arsenic and phthalates, are covered, but as Pfleger et al.^7^ noted, many relevant environmental factors remain unaddressed. Factors such as UV radiation, pesticides, ozone, noise, and pollen have not yet been incorporated, highlighting the challenge of comprehensively measuring EHL. Given that EHL is highly context-dependent, as described by Finn and O'Fallon,^5^ it remains unclear whether a single, generalized instrument can effectively capture the diverse environmental exposures and health risks across different communities.

A key finding of this systematic review is the significant heterogeneity in how EHL is operationalized, consistent with previous reviews by Gray^6^ and Pfleger et al.^7^ Two of the identified instruments use objective measurement methods, such as word recognition or numeracy tests, while the majority rely on self-assessments. Unlike objective assessments, self-assessment tools often reflect perceived rather than actual skills. This concern has been raised in studies of other health literacy domains, including cancer-specific and mental health literacy.^35,36^ At the same time, EHL extends beyond objective competencies, incorporating affective dimensions such as environmental concern.^14^ Since EHL aims to protect both individual health and the environment,^6^ a comprehensive assessment should include both knowledge-based and attitudinal components. Mixed-method approaches that integrate objective skills with subjective attitudes and motivations may therefore provide a more accurate representation of EHL.

More than half of the identified instruments were based on general HL concepts rather than EHL-specific definitions. Only three of the eight instruments focused exclusively on EHL without referencing broader HL models. The EHL scales and their Korean adaptation assess EHL through knowledge, attitudes, and behaviors.^24,25^ The PERHL scale follows a similar approach but extends beyond these dimensions by incorporating knowledge of phthalate exposure routes and health effects, attitudes toward regulation, protective behaviors, and perceived uncertainty.^28^ However, none of the identified instruments are based on conceptual models specifically designed for EHL, such as those by Finn and O'Fallon^5^ or Gray.^6^ Key dimensions are missing, including self-efficacy and the ability to develop preventive measures. Furthermore, all instruments focus exclusively on individual EHL, neglecting the community component essential for collective action on environmental health risks. Most instruments primarily assess knowledge and awareness of environmental risks, while social and community EHL dimensions remain largely unexamined.

In summary, these findings underscore the need to develop further EHL measurement tools that comprehensively capture both individual and collective dimensions of EHL.

### 4.2. Discussion of the Quality of the Instruments

According to the COSMIN criteria for good measurement properties,^19^ content validity is the most critical quality feature of a measurement instrument. A valid instrument should accurately reflect the construct, ensuring relevance, comprehensiveness, and comprehensibility; however, none of the included studies provided sufficient information to assess all 7 COSMIN criteria fully.

The content validity ratings were predominantly indetermined or sufficient, with one exception: the SA-EHL received an insufficient rating for comprehensiveness, as it does not cover the full spectrum of environmental health.^26^ However, the SA-EHL was specifically designed as a quick screening tool to assess basic EHL levels through word recognition. Rohlman et al.^26^ suggest that it is particularly useful for tailoring risk communication strategies after environmental disasters such as wildfires or hurricanes. Nevertheless, further validation—especially regarding structural validity and internal consistency—is needed before it can be widely recommended.

A key limitation in content validity is the limited involvement of the target population during concept elicitation and instrument development. Instead, content validity assessments relied on expert judgment and literature review only, which raises concerns about whether the instruments fully reflect the relevant EHL dimensions for their intended users. Since these tools are still in early development stages, future studies should address this gap by conducting focus groups or interview studies with peoples representing the intended target populations. For new scale developments, we also strongly recommend involving the target population throughout the whole process including concept elicitation, item development, and cognitive pretesting to improve relevance and validity.

Structural validity and internal consistency were the most thoroughly analyzed among the examined quality criteria. All eight instruments reported internal consistency data, but the results were highly heterogeneous. Many instruments exhibited insufficient reliability in specific dimensions, highlighting the need for further refinement. For example, the EHLI showed acceptable internal consistency overall, but its interactive EHL dimension did not meet the required threshold, indicating a need for revision. To improve structural validity, we recommend that researchers follow best-practice guidelines for scale development and validation, such as those outlined by Boateng et al.^37^ These guidelines suggest, for example, using the lack of satisfactory model fit as an opportunity to remove items with poor loadings and test alternative models (e.g., unidimensional vs. multidimensional models, higher-order factors) to identify the best-fitting solution. If necessary, one should also consider to revise items, to include new items and to conduct additional validation studies to improve the model.

Despite these analyses, key psychometric properties—such as intercultural validity, measurement error, and responsiveness—remain largely unexplored. This aligns with findings from Smith et al.,^36^ who identified similar gaps in mental health literacy assessment tools. Responsiveness, in particular, is crucial for instruments intended to evaluate interventions, as it determines whether an instrument can detect meaningful changes over time. Without such validation, it remains uncertain whether existing EHL instruments can reliably measure changes over time (e.g., improvements) resulting from educational, policy, or other interventions. A widely used method for assessing reliability is test-retest reliability,^37^ which evaluates how consistent a measure remains when administered to the same participants under similar conditions and without changes in the intended construct at different times. Beyond consistency, this approach to reliability ensures that a measure is able to capture a change in the construct and that changes in scores do not primarily reflect measurement error. In the case of EHL, evidence suggests that the construct is responsive to interventions, meaning that studies designed to shift EHL scores over time need to apply measures that have been proven to reveal sufficient test-retest reliability, but also to display sensitivity to (true) change. Notably, among the instruments reviewed, test-retest reliability was evaluated only for the IAPHL instrument.

Criterion validity, which assesses how well an instrument correlates with an established standard, was rated as insufficient for all four instruments compared with another measure. However, these findings should be interpreted cautiously. The COSMIN framework requires comparison with a recognized gold standard, which was not available in the studies examined. Instead, instruments were validated against HL tools, which measure related but distinct constructs. The strict correlation threshold of .70 may, therefore, be inappropriate, as HL and EHL, while overlapping, represent separate domains. Further research should compare EHL instruments and aim to establish a gold standard for EHL assessment.

Despite these limitations, the low correlation coefficients between HL and EHL scores provide valuable insights. They suggest that general HL does not necessarily equate to specific EHL.^22,26^ While HL focuses on individual health management, EHL encompasses additional competencies—including knowledge of environmental risks, protective behaviors, and the ability to interpret environmental health information. This underscores the need for dedicated EHL measurement tools rather than relying on broader HL assessments.

Overall, none of the reviewed instruments have been comprehensively validated, making it impossible to recommend any as a definitive tool for EHL measurement. The IAPHL instrument received the most sufficient ratings but still lacks critical validation data, particularly regarding content validity. Additional studies are necessary to refine existing instruments and improve their reliability and validity. Alternatively, future research could develop new instruments or scales based on established EHL models, such as those proposed by Finn and O’Fallon^5^ and Gray,^6^ to ensure a more comprehensive assessment of EHL across different populations.

### 4.3. Limitations

This study has some limitations that may affect the validity and generalizability of its findings.

First, limitations in study selection and search strategy may have influenced the comprehensiveness of the evidence base. While the full-text screening, risk of bias assessment, and quality appraisal were conducted independently by two reviewers, the initial title and abstract screening was performed by a single reviewer due to feasibility constraints. We acknowledge the risk of overlooking some findings. Restricting the literature search to three databases and to English- and German-language studies may have excluded valuable research from other databases as well as linguistic and geographical contexts.

The inclusion and exclusion criteria were relatively strict, potentially omitting relevant approaches to assessing EHL. For example, the Environmental Health Engagement Profile (EHEP)^34^ was excluded as it was not explicitly designed for EHL measurement, despite its relevance in assessing engagement with environmental health issues. Similarly, the Water Environmental Health Literacy (WEHL) instrument^38^ was omitted due to missing data on content and structural validity, even though it measures knowledge of toxic metal contamination in well water and includes self-efficacy assessment, a dimension often lacking in the included instruments. These exclusions may have limited the conceptual scope of the review.

Additionally, while the COSMIN guidelines provided a structured evaluation framework, they were originally designed for patient-reported outcome measures (PROMs) and may not be entirely appropriate for assessing instruments for EHL measurement. This is particularly relevant for criterion validity, where the comparison instruments were validated for HL rather than EHL, leading to weak correlations that did not meet the .70 threshold required by COSMIN. The lack of an EHL-specific gold standard further complicates the interpretation of these results.

The limited availability of psychometric data presents another challenge. For most instruments, assessments relied on a single study, limiting the reliability and validity of the quality ratings. Ideally, multiple independent studies should examine different psychometric properties to provide a more robust evaluation.^19^ Furthermore, heterogeneity among the included instruments—target populations, conceptual models, and methodological approaches—makes direct comparisons difficult.

Despite these limitations, this study provides a valuable first step in systematically assessing EHL measurement instruments and identifying areas for further refinement and validation.

## 5. Conclusion

This systematic review underscores the need for stronger psychometric evaluation of EHL measurement instruments to support both valid research and effective health promotion. Existing EHL instruments are still in early stages of development and display significant psychometric and conceptual limitations. Our review identifies the following gaps in the current literature and proposes clear directions for future research.

First, future research should prioritize enhancing measurement approaches by rigorously addressing content validity, reliability, and intercultural adaptation. Content validity can be improved by systematically involving representative samples of the target population at every stage of instrument development. In addition, comprehensive validation studies are needed to assess test-retest reliability, measurement invariance, sensitivity to change, and responsiveness, to ensure that instruments remain reliable over time and across contexts.

Second, there is a need to develop new instruments or scales grounded in established EHL models (e.g., Finn and O’Fallon,^5^ Gray^6^) to achieve more comprehensive and comparable assessments across populations. These tools should address neglected aspects such as self-efficacy and collective agency, which are often missing from current measures.

Third, beyond instrument refinement, future research should also explore the use of these tools in real-world interventions and policy programs, particularly for assessing and addressing environmental hazards. This would help ensure that measurement advances directly inform evidence-based practice and policy.

In summary, this review provides a clear roadmap for advancing EHL measurement: (1) strengthen psychometric rigor and content validity through stakeholder involvement; (2) develop multi-dimensional instruments based on established theoretical EHL models; and (3) apply and evaluate these tools in practical, policy-relevant contexts. Overall, this study provides a foundation for advancing practical, robust, theoretically grounded EHL measurement tools that capture the multi-dimensional nature of EHL.

## Supplemental Material

Supplemental Material - Measuring Environmental Health Literacy – A Systematic Review of Represented Dimensions and Psychometric Characteristics of Evaluated Instruments in an Emerging Field Supplemental Material for Measuring Environmental Health Literacy – A Systematic Review of Represented Dimensions and Psychometric Characteristics of Evaluated Instruments in an Emerging Field by Miriam Finkhäuser, Renée Madelaine Kuhrt, Marlene Muehlmann, Tobias Ihle, Silke Schmidt and Holger Muehlan in Environmental Health Insights.

## Data Availability

Data sharing not applicable to this article as no additional datasets were generated or analyzed during the current study. The original contributions presented in the study are included in the article and the Supplementary material. Further inquiries can be directed to the corresponding author.

## Appendix

### Abbreviations

AAPHL: Ambient Air Pollution Health Literacy
COSMIN: COnsenus-based Standards for the selection of health Measurement INstruments
EH: Environmental Health
EHEP: Environmental Health Engagement Profile
EHLI: Environmental Health Literacy Index
EHL: Environmental Health Literacy
EL: Environmental Literacy
HLS-EU-Q: European Health Literacy Survey Questionnaire
HL: Health Literacy
IAPHL: Indoor Air Pollution Health Literacy
K-EHEP: Korean Environmental Health Engagement Profile (Korean version of the EHEP)
K-EHL: Korean Environmental Health Literacy Scale
NVS: Newest Vital Sign
PERHL: Phthalate Environmental Reproductive Health Literacy
PRISMA: Preferred Reporting Items for Systematic Reviews and Meta-Analyses framework
PROMs: Patient-Reported Outcome Measures
SA-EHL: Short Assessment of Environmental Health Literacy
SAHL: Short Assessment of Health Literacy
UNESCO: United Nations Educational, Scientific and Cultural Organization
WEHL: Water Environmental Health Literacy
WELLS: Water Environmental Literacy Level Scale
WHO: World Health Organization
