# Supplementary material for: Measuring Environmental Health Literacy – A Systematic Review of Represented Dimensions and Psychometric Characteristics of Evaluated Instruments in an Emerging Field
Source: Environ Health Insights. 2026 Aug 4;20:11786302261462601. doi: 10.1177/11786302261462601 (PMC13438344; doi:10.1177/11786302261462601)
Supplement: Supplementary file 1 [file sj-pdf-1-ehi-10.1177_11786302261462601.pdf]

Measuring Environmental Health Literacy – A Systematic Review of represented dimensions and psychometric characteristics of evaluated instruments in an emerging field

Additional Files

Table S 1

Search strategies for the respective databases in English and German

Language	Search strategy
English<br>(for MEDLINE,<br>LIVIVO and PsycInfo)	(("environmental health literacy" OR ("environment*" AND "health litera*") OR "environmental health knowledge" OR "environmental health education") AND (instrument* OR tool OR survey* OR scale* OR assess* OR measure* OR inventory* OR questionnaire* OR interview*))
German<br>(for MEDLINE<br>and PsycInfo only)	(("environmental health literacy" OR ("environment*" AND "health literacy")) AND (*instrument* OR "tool" OR "survey*" OR "scale" OR "*assessment*" OR "*measure*" OR "*inventory*" OR "*questionnaire*" OR "*interview*"))

Table S 2

Inclusion and exclusion criteria for study selection

Inclusion criteria	Exclusion criteria
Development and/or validation of an instrument for assessing EHL	No assessment of EHL (e.g. only health literacy, environmental awareness, etc.)
Detailed information on the psychometric quality criteria of the instrument	Missing or insufficient information on quality criteria or no focus on the psychometric evaluation of the instrument
Publication language:<br>German or English	Other languages
Original work	Books, handbooks, reviews, meta-analyses, dissertations, commentaries

Table S 3

Results of the studies on content validity, structural validity and internal consistency

Instrument	Content validity	Structural validity	Internal consistency
AAPHL (Chen et al., 2023)	CVI, assessed by experts (n = 3):<br>Relevance = .97<br>Importance = .99<br>Uniqueness = .94	CFA (12-factor model):<br>RMSEA = .068, SRMR = .039,<br>CFI = .934, TLI = .902<br>Factor loadings: .61 - .88	Overall scale:<br>Cronbach's α = .93<br>Composite Reliability (CR) for the individual dimensions: .56 - .84
EHLI (Fiore et al., 2024)	Consensus process among the authors for the item selection	PCA: Factor loadings: .682 - .832<br>Explained variance:<br>Functional EHL (PC1): 35.3 %<br>Critical EHL (PC2): 14.4 %<br>Interactive EHL (PC3): 10.2 %<br>Total variance: 60.7 %<br>Eigenvalues: PC1: 4.595, PC2: 1.874, PC3: 1.424	Overall scale:<br>Cronbach's α = .808<br>Functional EHL:<br>Cronbach's α = .888<br>Critical EHL:<br>Cronbach's α = .795<br>Interactive EHL:<br>Cronbach's α = .471
EHL Scales (Lichtveld et al., 2019)	Visual validity: Review by experts (n = 3)		
(1) Air scale:		EFA: Factor loadings: -0.65 – 0.64; explained variance: knowledge (17.6 %), attitudes (13.3 %), behaviour (13.8 %)<br>CFA: RMSEA = .05	Cronbach's α = .70
(2) Food scale:		EFA: Factor loadings: .39 – .99; explained variance: knowledge (19.6 %), attitudes (38.1 %), behaviour (2.6 %)<br>CFA: RMSEA = .11	Cronbach's α = .67
(3) Water scale:		EFA: Factor loadings: -0.38 – 0.67; explained variance: knowledge (15.6 %), attitudes (24 %), behaviour (26.4 %)<br>CFA: RMSEA = .07	Cronbach's α = .63
(4) General UG scale:		EFA: factor loadings: .26 – .99; explained variance: attitudes (17.6 %), behaviour (12.6 %), knowledge (9.1 %)<br>CFA: RMSEA = .05	Cronbach's α = .70
IAPHL (Wu et al., 2022)	CVI, assessed by experts (n = 3):<br>Relevance = .97<br>Importance = .96<br>Clarity = .89	PCA<br>Five factors with eigenvalues > 1;<br>12 extracted components; Total variance explained: 71 %; Factor loadings: .55 – .79<br>CFA: SRMR = .055, RMSEA = .065, CFI = .868	Cronbach's α = .96

Instrument	Content validity	Structural validity	Internal consistency
K-EHL (Kwak & Kim, 2022)	CVI at item and scale level, assessed by experts (n = 3)<br><br>Scale level CVI: .92	EFA<br>Factor loadings: .32 – .64;<br>Explained variance: 15.83 % (factor 1), 11.36 % (factor 2); Total variance: 33.1 %;<br>Eigenvalues: 6.02 (factor 1), 4.32 (factor 2)<br>CFA: CFI = .901, TLI = .863, RMSEA = .08	Overall scale:<br>Cronbach's α = .83<br>Subscales:<br>Factor 1 (knowledge and attitudes):<br>Cronbach's α = .80<br>Factor 2 (behaviour):<br>Cronbach's α = .78
PERHL (Tomsho, Quinn, Adamkiewicz, et al., 2024)	Items were reviewed by experts for clarity, scope and redundancy	EFA<br>Factor loadings: 0.553 – 1.037;<br>Explained variance: 30.3 % (factor 1), 12.9 % (factor 2), 18 % (factor 3), 12.1 % (factor 4), 14 % (factor 5), 12.7 % (factor 6);<br>Eigenvalues: 11.64 (factor 1), 2.03 (factor 2), 0.74 (factor 3), 0.32 (factor 4), 0.15 (factor 5), 0.04 (factor 6)	Omega coefficients:<br>Factor 1: ω = .92<br>Factor 2: ω = .87<br>Factor 3: ω = .89<br>Factor 4: ω = .63<br>Factor 5: ω = .92<br>Factor 6: ω = .93
SA-EHL (Rohlman et al., 2022)	Review of the items on relevance and comprehensibility by environmental health professionals	EFA:<br>Number of extracted factors: 1 factor (eigenvalue > 1);<br>Confirmatory method:<br>Scree plot showed a clear drop in eigenvalue after the first factor	Cronbach's α = .56
WELLS (Irvin et al., 2019)	Evaluated by experts in the field of environmental health	EFA:<br>Number of extracted factors:<br>1 factor (eigenvalue > 1);<br>Confirmatory method:<br>Scree plot showed a clear drop in eigenvalue after the first factor	Cronbach's α = .51

Note. CFA: confirmatory factor analysis, CFI: comparative fit index, CVI: content validity index, EFA: exploratory factor analysis, PCA: principal component analysis, RMSEA: root mean square error of approximation, SRMR: standardised root mean square residual, TLI: Tucker-Lewis index, UG: environmental health.

AAPHL: Ambient Air Pollution Health Literacy Instrument, EHLI: Environmental Health Literacy Index, EHL Scales: Environmental Health Literacy Scales, IAPHL: Indoor Air Pollution Health Literacy Instrument, K-EHL: Korean version of the Environmental Health Literacy Scale, PERHL: Phthalate Environmental Reproductive Health Literacy Scale, SA-EHL: Short Assessment of Environmental Health Literacy, WELLS: Water Environmental Literacy Level Scale.

Table S 4

Results of the studies on retest reliability, criterion validity and construct validity

Instrument	Retest reliability	Criterion validity	Construct validity (hypothesis testing)
EHLI (Fiore et al., 2024)	-	Correlations:<br>EHLI and functional *health literacy* (HL):<br>Spearman's ρ = .183, $p < .001$<br>EHLI and level of adoption of pro-ecological behaviours (APEB):<br>Spearman's ρ = .248, $p < .001$<br>AUC: .643 ($p < .001$)	-
IAPHL (Wu et al., 2022)	ICC = .94<br>Time interval between measurements: 4 weeks	-	Discriminant validity:<br>Hypothesis:<br>IAPHL scores and weekly television viewing time are only weakly correlated<br>Results:<br>Spearman's ρ = -0.14, $p < .001$
K-EHL (Kwak & Kim, 2022)	-	Correlation between K-EHL and K-EHEP:<br>$r = .67$, $p < .01$	-
PERHL (Tomsho, Quinn, Adamkiewicz, et al., 2024)	-	-	Hypothesis: Correlation between PERHL scores and avoidance of products containing phthalates (self-assessment)<br>Result: Higher total scores on the PERHL scale are associated with a lower probability of avoiding products containing phthalates (OR = 0.63),<br>OR for the individual dimensions: 0.14 – 21.21
PERHL (Tomsho, Quinn, Preston, et al., 2024)	-	-	Hypothesis: Higher scores on the PERHL scale are associated with lower concentrations of phthalates in the urine samples<br>Result: Increase of one point in the total score of the PERHL scale significantly associated with a decrease of -7.2% in the phthalate concentrations

Instrument	Retest reliability	Criterion validity	Construct validity (hypothesis testing)
SA-EHL (Rohlman et al., 2022)	-	Correlation between SAHL and SA-EHL: r = .11, p. < .001<br>Kappa value: .0018 (p = .18), 10.8 % agreement<br>Bland-Altman plot: no pattern of agreement pattern between the both scales	-
WELLS (Irvin et al., 2019)	-	Correlation between WELLS and NVS: = .47, p < .001<br>Kappa value: .29 (p < .03), 74 % agreement<br>Bland-Altman plot: Mean value of the difference: 0.012 (*SD* = 1.16), 95% Limits of agreement: -1.15 – 1.18	Hypothesis: positive correlation between experience in dealing with wells and WELLS scores<br>Result: could not be confirmed

Note. APEB: Adoption of pro-environmental behaviours, AUC: Area under the curve, ICC: Intraclass correlation, K-EHEP: Korean version of the Environmental Health Engagement Profile, NVS: Newest Vital Sign, OR: Odds Ratio, SAHL: Short Assessment of Health Literacy.

Table S 5

Criteria for the evaluation of study results on content validity

Criteria		Requirements for "sufficient" (+) rating
Relevance		
R1.	Are the included items relevant for the construct of interest?	The construct is clearly described<br>AND<br>The origin of the construct is clearly described<br>AND<br>There is evidence from concept elicitation, literature or experts that at least 85% of the items refer to the construct of interest<br>AND<br>The items should be specific for the construct of interest, i.e. they should not measure a concurrent but separate construct<br>AND<br>No unnecessary items should be included (e.. too many items)
R2	Are the included items relevant for the target population of interest?	The target population is clearly described<br>AND<br>A representative sample was involved in the concept elicitation
R3.	Are the included items relevant for the context of use of interest?	The context of use is clearly described<br>AND<br>Experts were involved in the development of the instrument
R4.	Are the response options appropriate?	A justification is provided for the response options
Comprehensiveness		
C1.	Are all key concepts included?	Saturation was reached in the concept development phase
Comprehensibility		
C2.	Are the instructions of the instrument understood by the target population as intended?	Participants were asked about the comprehensibility of the instructions in a pilot study<br>AND<br>Problems were adequately addressed
C3.	Are the items and response options understood by the target population as intended?	Participants were asked about the comprehensibility of the items and the response options (including the wording) in a pilot study<br>AND<br>Problems were adequately addressed

Note. The content validity assessment criteria listed in this table are based on the COSMIN *Criteria for good measurement properties* and have been adapted for the specific context of this review. Further information on the full criteria can be found in the COSMIN manual (Mokkink et al., 2024).

Table S 6

Criteria for evaluating the study results for further quality criteria

Quality criteria	Requirements for "sufficient" (+) rating
Structural validity	EFA / PCA (exploratory factor analysis / principal component analysis):<br>Factor loadings of the individual items on their factor ≥ .30<br>AND<br>A maximum of 10 % of the items load on more than one factor<br>AND<br>Explained variance ≥ 50 % and the structure corresponds to the theory of the construct to be measured OR the results in the scree plot or according to the Kaiser criterion (eigenvalues >1) correspond to the theory of the construct to be measured<br><br>CFA (confirmatory factor analysis):<br>CFI or TLI or comparable measure > .95 OR RMSEA < .06 OR SRMR < .08
Internal consistency	Cronbach's α or a comparable measure ≥ .70
Retest reliability	ICC or (weighted) kappa or Pearson/Spearman correlation ≥ .70
Criterion validity	Correlation with a gold standard* ≥ .70 OR AUC ≥ .70
Construct validity (hypothesis testing)	≥ 75 % of the results agree with the predefined hypotheses

Note. The criteria listed in this table for assessing the quality criteria are based on the COSMIN *Criteria for good measurement properties*. The quality criteria *intercultural validity*, *measurement error* and *responsiveness* are not shown in this table, as none of the included studies reported information on these. Further information on the complete criteria can be found in the COSMIN manual (Mokkink et al., 2024).
AUC: Area under the curve, CFI: Comparative Fit Index, ICC: Intraclass correlation, RMSEA: Root Mean Square Error of Approximation, SRMR: Standardised Root Mean Square Residual, TLI: Tucker-Lewis Index.
* In this paper, the term "gold standard" was also applied to comparisons with instruments that measure related constructs, such as *health literacy*, but do not necessarily represent an established gold standard for measuring EHL. Given the current absence of universally accepted standards for EHL measurement instruments, this assessment category was broadened accordingly.

Table S 7

Excluded studies and reasons for exclusion

Reference	Reason for exclusion
Bert, F., Gea, M., Previti, C., Massocco, G., Lo Moro, G., Scaioli, G., Schilirò, T., & Siliquini, R. (2023). The environmental health literacy of Italian general population: The SPeRA cross-sectional study. *International Journal of Environmental Research and Public Health*, *20*(5), 4486. https://doi.org/10.3390/ijerph20054486	(2)
Binder, A. R., May, K., Murphy, J., Gross, A., & Carlsten, E. (2022). Environmental health literacy as knowing, feeling, and believing: Analyzing linkages between race, ethnicity, and socioeconomic status and willingness to engage in protective behaviours against health threats. *International Journal of Environmental Research and Public Health*, *19*(5), 2701. https://doi.org/10.3390/ijerph19052701	(2)
Bogar, S., Szabo, A., Woodruff, S., & Johnson, S. (2017). Urban youth knowledge and attitudes regarding lead poisoning. *Journal of Community Health: The Publication for Health Promotion and Disease Prevention*, *42*(6), 1255-1266. https://doi.org/10.1007/s10900-017-0378-8	(1)
Boronow, K. E., Cohn, B., Havas, L., Plumb, M., & Brody, J. G. (2023). The effect of individual or study-wide report-back on knowledge, concern, and exposure-reducing behaviours related to endocrine-disrupting chemicals. *Environmental Health Perspectives*, *131*(9), 97005. https://doi.org/10.1289/EHP12565	(2)
Brewer, D., Bellamy, H., Hoover, A., Koempel, A., & Gaetke, L. (2019). Nutrition and environmental pollution extension curriculum improved diet-related behaviours and environmental health literacy. *Environmental Health Insights*, *13*. https://doi.org/10.1177/1178630219836992	(2)
Ciarloni, R., & Newbold, K. B. (2023). Air pollution health literacy among active commuters in Hamilton, Ontario. *International Journal of Environmental Research and Public Health*, *20*(13), 6282. https://doi.org/10.3390/ijerph20136282	(2)
Davis, L. F., Ramirez-Andreotta, M. D., McLain, J. E. T., Kilungo, A., Abrell, L., & Buxner, S. (2018). Increasing environmental health literacy through contextual learning in communities at risk. *International Journal of Environmental Research and Public Health*, *15*(10), 2203. https://doi.org/10.3390/ijerph15102203	(2)
Dellinger, M. J., Lyons, M., Clark, R., Olson, J., Pingatore, N., & Ripley, M. (2019). Culturally adapted mobile technology improves environmental health literacy in Laurentian, Great Lakes Native Americans (Anishinaabeg). *Journal of Great Lakes Research*, *45*(5), 969-975. https://doi.org/10.1016/j.jglr.2019.07.003	(2)
Dellinger, M. J., Pingatore, N., Chelius, T., Visotcky, A., Sparapani, R., & Ripley, M. (2022). Environmental health literacy for Anishinaabe (Great Lakes Native American) fish consumers: A randomised control trial. *Environmental Research*, *212*, 113335. https://doi.org/10.1016/j.envres.2022.113335	(2)
Dixon, J. K., Hendrickson, K. C., Ercolano, E., Quackenbush, R., & Dixon, J. P. (2009). The Environmental Health Engagement Profile: What people think and do about environmental health. *Public Health Nursing*, *26*(5), 460-473. https://doi.org/10.1111/j.1525-1446.2009.00804.x	(1)
Gray, K. M., Triana, V., Lindsey, M., Richmond, B., Hoover, A. G., & Wiesen, C. (2021). Knowledge and beliefs associated with environmental health literacy: A case study focused on toxic metals contamination of well water. *International Journal of Environmental Research and Public Health*, *18*(17), 9298. https://doi.org/10.3390/ijerph18179298	(2)
Hou, W.-H., Huang, Y.-C., Lu, C.-Y., Chen, I.-C., Lee, P.-C., Lin, M.-Y., Wang, Y.-C., Sulistyorini, L., & Li, C.-Y. (2021). A national survey of ambient air pollution health literacy among adult residents of Taiwan. *BMC Public Health*, *21*(1), 1604. https://doi.org/10.1186/s12889-021-11658-z	(2)
Maaoui, F., Moumni, I., Arboix-Calas, F., Safra, I., & Menif, S. (2022). Emotional and behavioural attitudes of Tunisian youth towards childhood leukemia: Health education and primary prevention in perspective. *BMC Public Health*, *22*(1), 2105. https://doi.org/10.1186/s12889-022-14596-6	(2)
Madrigal, D., Claustro, M., Wong, M., Bejarano, E., Olmedo, L., & English, P. (2020). Developing youth environmental health literacy and civic leadership through community air monitoring in Imperial County, California. *International Journal of Environmental Research and Public Health*, *17*(5), 1537. https://doi.org/10.3390/ijerph17051537	(2)
Pansakun, N., Naksen, W., Boonchieng, W., Ong-artborirak, P., & Prapamontol, T. (2024). Environmental health literacy regarding fine particulate matter and related factors among village health volunteers in upper northern Thailand. *Journal of Preventive Medicine and Public Health*, *57*(2), 138-147. https://doi.org/10.3961/jpmph.23.434	(2)
Pfleger, E., Drexler, H., & Lutz, R. (2024). Health literacy and environmental risks focusing air pollution: Results from a cross-sectional study in Germany. *International Journal of Environmental Research and Public Health*, *21*(3), 366. https://doi.org/10.3390/ijerph21030366	(1)
Quintyne, K. I., & Kelly, C. (2023). Knowledge, attitudes, and perception of air pollution in Ireland. *Public Health in Practice, 6,* 100406. https://doi.org/10.1016/j.puhip.2023.100406	(1)

Reference	Reason for exclusion
Ramirez-Andreotta, M. D., Brody, J. G., Lothrop, N., Loh, M., Beamer, P. I., & Brown, P. (2016). Improving environmental health literacy and justice through environmental exposure results communication. *International Journal of Environmental Research and Public Health*, *13*(7), 690. https://doi.org/10.3390/ijerph13070690	(2)
Ratnapradipa, D., Middleton, W. K., Wodika, A. B., Brown, S. L., & Preihs, K. (2015). What does the public know about environmental health? A qualitative approach to refining an environmental health awareness instrument. *Journal of Environmental Health*, *77*(8), 22-29.	(2)
Raufman, J., Blansky, D., Lounsbury, D. W., Mwangi, E. W., Lan, Q., Olloquequi, J., & Hosgood, H. D. (2020). Environmental health literacy and household air pollution-Associated symptoms in Kenya: A cross-sectional study. *Environmental Health*, *19*(1), 89. https://doi.org/10.1186/s12940-020-00643-5	(2)
Riley, K. W., Burke, K., Dixon, H., Holmes, D., Calero, L., Barton, M., Miller, R. L., Bramer, L. M., Waters, K. M., Anderson, K. A., Herbstman, J., & Rohlman, D. (2024). Development and outcomes of returning polycyclic aromatic hydrocarbon exposure results in the Washington Heights, NYC Community. *Environmental Health Insights*, *18*. https://doi.org/10.1177/11786302241262604	(2)
Rochester, J. R., Kwiatkowski, C. F., Neveux, I., Dabe, S., Hatcher, K. M., Lathrop, M. K., Daza, E. J., Eskenazi, B., Grzymski, J. J., & Hua, J. (2024). A personalised intervention to increase environmental health literacy and readiness to change in a northern Nevada population: Effects of environmental chemical exposure report-back. *International Journal of Environmental Research & Public Health*, *21*(7), 905. https://doi.org/10.3390/ijerph21070905	(2)
Simonds, V. W., Kim, F. L., LaVeaux, D., Pickett, V., Milakovich, J., & Cummins, J. (2019). Guardians of the living water: Using a health literacy framework to evaluate a child as change agent intervention. *Health Education & Behaviour*, *46*(2), 349-359. https://doi.org/10.1177/1090198118798676	(2)
Stanifer, S., Hoover, A. G., Rademacher, K., Rayens, M. K., Haneberg, W., & Hahn, E. J. (2022). Citizen science approach to home radon testing, environmental health literacy and efficacy. *Citizen Science: Theory and Practice*, *7*(1), 26. https://doi.org/10.5334/cstp.472	(2)
Tomsho, K. S., Polka, E., Chacker, S., Queeley, D., Alvarez, M., Scammell, M. K., Emmons, K. M., Rudd, R. E., & Adamkiewicz, G. (2022). Characterising the environmental health literacy and sensemaking of indoor air quality of research participants. *International Journal of Environmental Research & Public Health*, *19*(4), 2227. https://doi.org/10.3390/ijerph19042227	(1)
Wang, J., Ding, X., Li, D., & Li, S. (2022). The impact of organisational support, environmental health literacy on farmers' willingness to participate in rural living environment improvement in China: Exploratory analysis based on a PLS-SEM model. *Agriculture*, *12*(11), 1798. https://doi.org/10.3390/agriculture12111798	(2)
Wong, C., Wu, H.-C., Cleary, E. G., Patton, A. P., Xie, A., Grinstein, G., Koch-Weser, S., & Brugge, D. (2019). Visualising air pollution: Communication of environmental health information in a Chinese immigrant community. *Journal of Health Communication*, *24*(4), 339-358. https://doi.org/10.1080/10810730.2019.1597949	(2)
Zhao, Y., Sheng, Y., Zhou, J., Wang, H., Chilufya, M. M., Liu, X., Mohamed, A. O., Han, J., & Qu, C. (2022). Influencing factors of residents' environmental health literacy in Shaanxi province, China: A cross-sectional study. *BMC Public Health*, *22*(1), 114. https://doi.org/10.1186/s12889-022-12561-x	(2)

Note. 1eason for exclusion (1): no specific instrument for assessing EHL, reason for exclusion (2): insufficient information on quality criteria or lack of psychometric evaluation of an instrument.

Table S 8

Risk of Bias Assessment

QualSyst	Chen et al. (2023)	Fiore et al. (2024)	Irvin et al (2019)	Kwak & Kim (2022)	Lichtveld et al (2019)	Rohlman et al. (2022)	Tomsho, Quinn, Adamkiewicz, et al. (2024)	Tomsho, Quinn, Preston, et al. (2024)	Wu et al. (2022)
Question / objective sufficiently described? (no NA possible)	2	2	2	2	2	2	2	2	2
Study design evident and appropriate? (no NA possible)	2	1	2	2	2	2	2	2	2
Method of subject/comparison group selection or source of information/input variables described and appropriate?	2	2	0	2	2	2	2	0	2
Subject (and comparison group, if applicable) characteristics sufficiently described? (no NA possible)	2	1	1	2	2	2	2	2	2
If interventional and random allocation was possible, was it described?	NA	NA	NA	NA	NA	NA	NA	NA	NA
If interventional and blinding of investigators was possible, was it reported?	NA	NA	NA	NA	NA	NA	NA	NA	NA
If interventional and blinding of subjects was possible, was it reported?	NA	NA	NA	NA	NA	NA	NA	NA	NA

QualSyst	Chen et al. (2023)	Fiore et al. (2024)	Irvin et al (2019)	Kwak & Kim (2022)	Lichtveld et al (2019)	Rohlman et al. (2022)	Tomsho, Quinn, Adamkiewicz, et al. (2024)	Tomsho, Quinn, Preston, et al. (2024)	Wu et al. (2022)
Outcome and (if applicable) exposure measure(s) well defined and robust to measurement / misclassification bias? Means of assessment reported?	1	2	0	2	2	2	2	2	1
Sample size appropriate?	2	2	1	2	1	2	1	1	2
Analytic methods described/justified and appropriate?	2	1	1	2	2	2	2	2	1
Some estimate of variance is reported for the main results?	2	2	1	2	2	2	2	2	2
Controlled for confounding?	NA	NA	NA	NA	NA	NA	NA	NA	NA
Results reported in sufficient detail? (no NA possible)	2	2	0	2	1	2	2	2	2
Conclusions supported by the results? (no NA possible)	2	2	2	2	1	1	2	2	2
Mean	1,90	1,70	1,00	2,00	1,70	1,90	1,90	1,70	1,80

Note. Two raters performed a risk-of-bias assessment using the Checklist for assessing the quality of quantitative studies (Kmet et al., 2004). 2 = yes, 1 = partial and 0 = no.
